# Differential Effects of Saturated and Unsaturated Fatty Acids on Colorectal Cancer via IDO1 Signaling

**DOI:** 10.1002/cam4.71644

**Published:** 2026-03-08

**Authors:** Shulin Zhang, Jing Zhang, Jiaqi Chen, Abdel Nasser B. Singab, Guang Yang, Jiaxuan Li, Peiyao Li, Songlin Wu, Di Zhao, Junhua Sun, Lifen Gao, Guimei Lin, Guanying Yu, Yanfeng Iv, Hongbo Wang, Lin Yuan, Lan Ye

**Affiliations:** ^1^ Cheeloo College of Medicine Shandong University Jinan China; ^2^ Department of Gastroenterology, the Second Hospital of Shandong University Jinan China; ^3^ Department of Gastroenterology, Beijing Tsinghua Changgung Hospital, School of Clinical Medicine Tsinghua University Beijing China; ^4^ The First Clinical Medical College of Shandong University of Traditional Chinese Medicine Jinan China; ^5^ Cancer Center The Second Hospital of Shandong University Jinan China; ^6^ Department of Pharmacognosy Ain‐Shams University Cairo Egypt; ^7^ Centre of Drug Discovery Research and Development Ain Shams University Cairo Egypt; ^8^ Key Laboratory for Experimental Teratology of Ministry of Education, Shandong Key Laboratory of Infection and Immunity, and Department of Immunology, School of Basic Medical Sciences, Cheeloo College of Medicine Shandong University Jinan China; ^9^ Key Laboratory for Technology Research and Evaluation of Drug Products, School of Pharmaceutical Sciences, Cheeloo College of Medience Shandong University Jinan China; ^10^ Department of Gastrointestinal Surgery Central Hospital Affiliated to Shandong First Medical University Jinan China; ^11^ Department of Colorectal Surgery, the Second Hospital of Shandong University Jinan China; ^12^ Key Laboratory of Computing Power Network and Information Security, Ministry of Education, Shandong Computer Science Center Qilu University of Technology (Shandong Academy of Sciences) Jinan China; ^13^ Shandong Engineering Research Center of Big Data Applied Technology, Faculty of Computer Science and Technology Qilu University of Technology (Shandong Academy of Sciences) Jinan China; ^14^ Shandong Provincial Key Laboratory of Industrial Network and Information System Security Shandong Fundamental Research Center for Computer Science Jinan China

**Keywords:** colorectal cancer, fatty acids, gut microbiota, high‐fat diet, IDO1

## Abstract

**Background:**

Colorectal cancer (CRC) incidence has risen significantly in China, potentially linked to dietary Westernization and increased consumption of high‐fat diets (HFD).

**Methods:**

This study examines the differential effects of various fatty acids—saturated (palmitic acid, PA), monounsaturated (oleic acid, OA), and polyunsaturated (arachidonic acid, AA; docosahexaenoic acid, DHA)—on CRC progression, focusing on the IDO1/AhR signaling pathway.

**Results:**

Clinical data indicate that CRC patients exhibit elevated serum lipid levels, with PA promoting cell proliferation, migration, and invasion more strongly than unsaturated fatty acids. The pro‐tumorigenic effects of PA are enhanced in the presence of lipopolysaccharide (LPS), suggesting an interaction between diet and gut microbiota. In vivo experiments corroborate that a high‐PA diet significantly elevates tumor growth and IDO1 expression compared to DHA. Mechanistic analyses reveal that PA and LPS co‐stimulation activates the IDO1‐AhR‐PI3K/Akt‐NF‐κB pathway, which is implicated in CRC progression.

**Conclusions:**

These findings suggest that saturated fatty acids, particularly PA, may exacerbate CRC development risk through metabolic dysregulation, highlighting the potential of IDO1 as a biomarker for CRC associated with HFD. Reducing dietary saturated fat intake may thus be a viable strategy for CRC prevention.

## 1 Introduction

Colorectal cancer (CRC) is one of the most prevalent malignant tumors, with both incidence and mortality rates steadily increasing in China. The 2020 China Cancer Statistics Report ranked CRC as the second most common and the fifth deadliest among all malignancies. In 2020 alone, China recorded 555,000 new cases and 286,000 deaths due to CRC, underscoring the substantial risk it posed to public health and human life [1]. Notably, the incidence is higher in urban areas, likely associated with Westernized lifestyles and diets. High‐fat diets (HFDs), rich in saturated and unsaturated fatty acids, have been implicated in CRC pathogenesis [2]. However, the specific roles of different fatty acids remain poorly understood.

The typical Western diet (WD) comprises approximately 50% carbohydrates, 15% protein, and 35% fat, with the fat content exceeding the 25%–35% upper limit recommended by dietary guidelines [3]. A high‐fat diet provides abundant fatty acids that are hydrolyzed in the intestine, absorbed by epithelial cells, resynthesized into lipids such as triglycerides and cholesterol, and released into the bloodstream to participate in lipid metabolism. It is widely accepted that a high‐fat diet, particularly one rich in fatty acids, elevates the risk of colorectal cancer (CRC) [4]. Excessive dietary fat intake induces hyperlipidemia, a systemic lipid metabolism disorder characterized by elevated serum levels of total cholesterol (TC), triglycerides (TG), and/or low‐density lipoprotein (LDL‐C) and reduced levels of high‐density lipoprotein (HDL‐C) [5]. Rising dietary fat intake has elevated blood lipid levels among Chinese residents, making hyperlipidemia an increasingly prevalent chronic disease and a growing burden on healthcare resources [6].

Research has demonstrated that individuals adhering to a long‐term Western diet exhibit elevated plasma levels of kynurenine (Kyn), with diet playing a pivotal role in regulating host‐gut microbiota interactions [7, 8]. Multi‐omics studies have confirmed that a high‐fat diet disrupts gut microbiota‐colonocytes interactions, resulting in aberrant tryptophan (Trp)‐Kyn metabolism and metabolic reprogramming within the colon [9, 10]. Indoleamine 2, 3‐dioxygenase‐1 (IDO1) serves as the rate‐limiting enzyme in Trp metabolism, catalyzing its conversion into the end product Kyn [11]. This metabolic process depletes Trp in the local microenvironment, produces cytotoxic metabolites, and disrupts immune cell functions within the tissue microenvironment. These effects collectively induce immunosuppression, facilitating tumor immune evasion [12]. The aryl hydrocarbon receptor (AhR) is a transcription factor with critical regulatory roles in the metabolism of environmental polycyclic aromatic compounds, immune response modulation, circadian rhythm regulation, reproduction, and oxidative stress management, achieved through the transcriptional regulation of downstream genes [13]. Its endogenous ligands include metabolites derived from Typ, heme, and arachidonic acid. Within the tumor microenvironment, AhR interacts with ligands to enhance cell proliferation and suppress apoptosis, thereby contributing to tumor progression [14]. In CRC, Kyn, a metabolic product of IDO1, serves as a ligand for AhR, leading to the activation of the AhR signaling pathway. AhR also functions as a transcription factor for the PI3K/Akt signaling pathway, facilitating its activation and ultimately upregulating the NF‐κB expression [15]. High‐fat diet has been shown to upregulate the IDO1 expression in the intestinal environment by altering the gut microbiota, leading to disruptions in the Trp‐Kyn metabolic pathway [16].

High‐fat diets are rich in fatty acids, classified as saturated (SFA), monounsaturated (MUFA), or polyunsaturated (PUFA), with PUFAs further divided into Ω‐3 and Ω‐6 based on double‐bond position. Although high‐fat diets are linked to CRC, the effects of specific fatty acids remain unclear. To address this, the present study focuses on four representative fatty acids: palmitic acid (PA), oleic acid (OA), arachidonic acid (AA), and docosahexaenoic acid (DHA), corresponding to SFA, MUFA, Ω‐3 PUFA, and Ω‐6 PUFA, respectively. This study aims to elucidate the differential effects of these fatty acids on CRC progression and to investigate the involvement of the IDO1/AhR signaling pathway.

## 2 Results

### 2.1 Elevated Serum Lipid Levels Are Associated With Colorectal Cancer

This study initially examined whether serum lipid level in humans is associated with CRC. The characteristics of all participants are summarized in (Table 1). There were no significant differences in age, sex, comorbidities (including hypertension, coronary heart disease, and diabetes) or smoking and alcohol consumption history between participants with CRC and those without CRC. The serum lipid profiles of participants with and without CRC are detailed (Table 2). Participants with CRC exhibited significantly higher serum levels of total cholesterol (TC), low‐density lipoprotein cholesterol (LDL‐C), apolipoprotein A1 (APOA1), apolipoprotein B (APOB), lipoprotein a (LPa), and non‐esterified fatty acid (NEFA) compared to non‐CRC participants. In contrast, HDL‐C level was significantly lower in CRC patients. No significant difference was found in triacylglycerol (TG) or small dense low‐density lipoprotein cholesterol (sdLDL‐C) levels between the two groups. These findings indicate that CRC patients exhibit altered lipid metabolism, characterized by elevated serum lipid levels, which may play an important role in the pathogenesis of colorectal cancer.

**TABLE 1:** Participants characteristics.

		All participants (*n* = 302)	Without colorectal cancers (*n* = 156)	Colorectal cancer (*n* = 146)	*p*
Age (years) *		58.01 ± 12.63	58.90 ± 8.84	57.32 ± 9.90	0.146
Sex	Male	175	90	85	0.926
	Female	127	66	61	
Hypertension	Yes	39	18	21	0.461
	No	263	138	125	
Coronary heart disease	Yes	28	13	15	0.561
	No	274	143	131	
Diabetes	Yes	33	16	17	0.699
	No	269	140	129	
Smoking history	Yes	35	15	20	0.268
	No	267	141	126	
Alcohol consumption history	Yes	40	22	18	0.650
	No	262	134	128	

* The values were presented as mean ± standard deviation.

**TABLE 2:** Serum lipid level of all participants with and without colorectal cancer.

Characteristics	All participants (*n* = 302)	Without colorectal cancers (*n* = 156)	Colorectal cancer (*n* = 146)	*p*
TC (mmol/L)	4.30 (3.79 ~ 5.04)	4.24 (3.45 ~ 4.81)	4.54 (4.00 ~ 5.15)	< 0.01**
TG (mmol/L)	1.00 (0.73 ~ 1.37)	1.07 (0.69 ~ 1.38)	1.13 (0.76 ~ 1.36)	0.27
HDL‐C (mmol/L)	1.19 (1.00 ~ 1.48)	1.10 (0.87 ~ 1.24)	1.40 (1.15 ~ 1.56)	< 0.01**
LDL‐C (mmol/L)	2.57 (2.07 ~ 3.06)	2.55 (1.97 ~ 2.97)	2.83 (2.23 ~ 3.10)	< 0.05*
APOA1 (g/L)	1.31 (1.14 ~ 1.53)	1.18 (1.02 ~ 1.36)	1.46 (1.29 ~ 1.62)	< 0.01**
APOB (g/L)	0.93 (0.77 ~ 1.13)	0.91 (0.77 ~ 1.08)	0.98 (0.79 ~ 1.15)	< 0.05*
LPa (nmol/L)	31.40 (11.50 ~ 66.83)	21.15 (9.25 ~ 54.83)	39.80 (15.70 ~ 74.50)	< 0.01**
sdLDL‐C (mmol/L)	0.59 (0.43 ~ 0.84)	0.58 (0.42 ~ 0.82)	0.63 (0.43 ~ 0.86)	0.28
NEFA (mmol/L)	0.54 (0.39 ~ 0.72)	0.56 (0.38 ~ 0.70)	0.62 (0.39 ~ 0.73)	< 0.01**

Abbreviations: APO, apolipoprotein; HDL‐C, high density lipoprotein cholesterol; LDL‐C, low density lipoprotein cholesterol; LPa, lipoprotein a; NEFA, non‐esterified fatty acid; sdLDL‐C, small dense low‐density lipoprotein cholesterol; TC, total cholesterol; TG, triacylglycerol.

* The values were presented as medians (upper quartiles~lower quartiles). *p＜0.05; **p＜0.01.

### 2.2 Saturated Fatty Acids (SFAs) and Lipopolysaccharide (LPS) Promote the Proliferation, Migration and Invasion of Colon Cancer In Vitro and In Vivo

**FIGURE 1:**
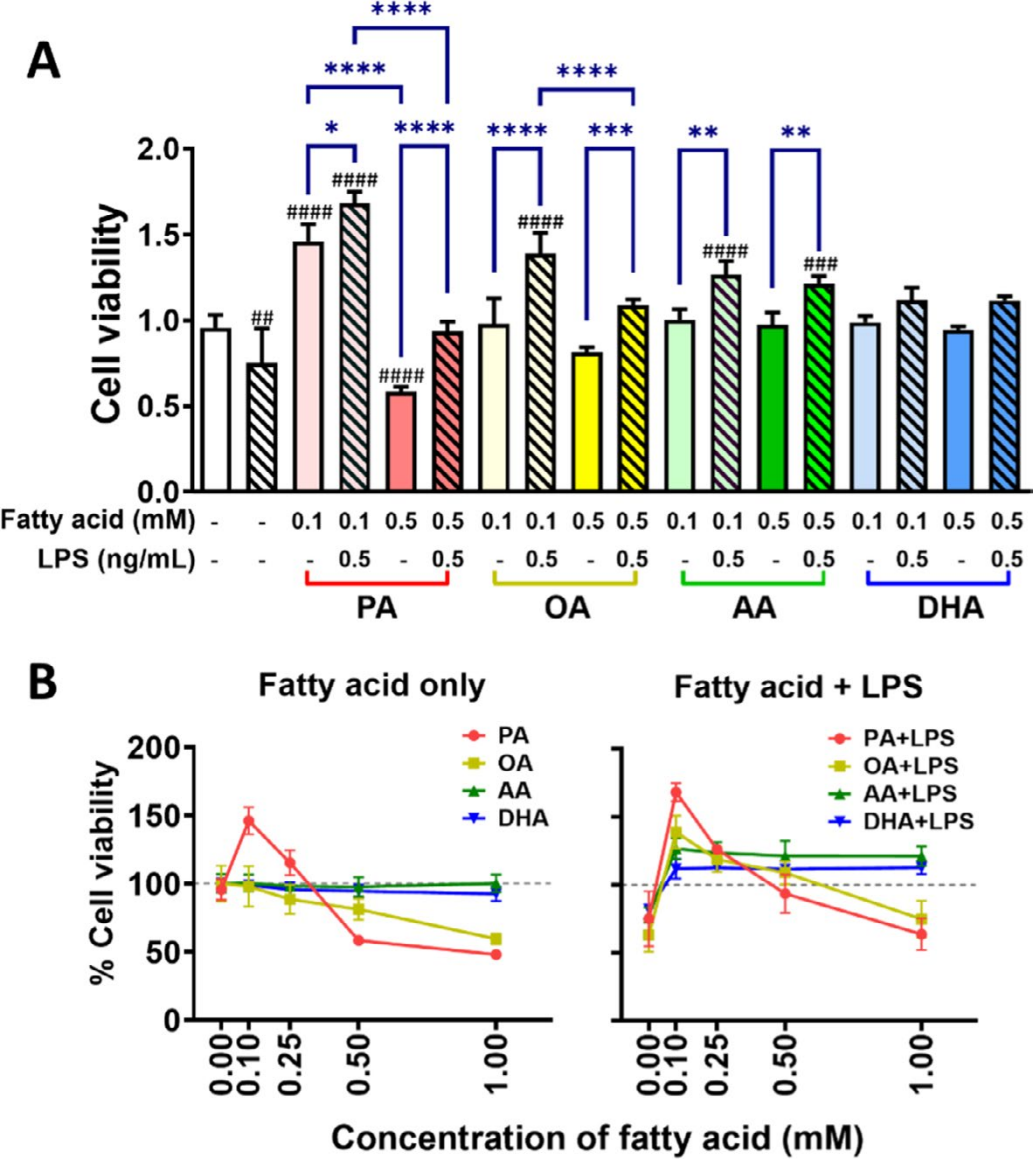
Effects of Fatty Acids and LPS on Colon Cancer Cell Proliferation In Vitro. HT‐29 colon cancer cells were cultured with fatty acids (PA, OA, AA, DHA; 0.1–1.0 mM) and/or LPS (0.5 ng/mL) for 24 h. Cell viability was measured using Cell Counting Kit‐8 (CCK‐8) assay. Results are presented as (A) Bar graph and (B) line graph to illustrate differences between groups. Data presented as the mean ± SD; *n* = 6 samples per group. ^#^ *p* < 0.05; ^##^ *p* < 0.01; ^###^ *p* < 0.001; ^####^ *p* < 0.0001 compared with vehicle groups and *p < 0.05; **p < 0.01; ***p < 0.001; ****p < 0.0001 by 1‐way ANOVA with Dunnett's multiple‐comparisons test and Bartlett's test of equal variances.

Given the observed association between elevated serum non‐esterified fatty acid (NEFA) levels and CRCs we further investigated whether fatty acids influence CRC progression. The colonrectal cancer cell line HT‐29 was utilized to evaluate the effects of various fatty acids on cellular proliferation, migration and invasion. Representative fatty acids, including palmitate acid (PA; saturated fatty acid), oleic acid (OA; monounsaturated fatty acid), arachidonic acid (AA; polyunsaturated fatty acid) and docosahexaenoic acid (DHA; polyunsaturated fatty acid), were selected for analysis. Lipopolyaccharide, a major product of Gram‐negative enteric microbiota, has been shown to increase in circulation following high‐fat diet (HFD) consumption [17]. To mimic the in vivo effect of fatty acid, LPS was used to mimic the gut microbiota in vitro. HT‐29 cells were treated with PA (0.1–1.0 mM) and/or LPS (0.5 ng/mL) for 24 h to investigate the underlying mechanisms. OA, AA and DHA were applied using the same protocol. Cell viability was assessed using Cell Counting Kit‐8 assay. LPS alone suppressed HT‐29 cell proliferation at 0.5 ng/mL compared to the vehicle control (*p* < 0.01). PA alone promoted proliferation at 0.1–0.25 mM (*p* < 0.0001), and this effect was further enhanced in the presence of LPS (*p* < 0.05). At the 0.5–1.0 mM, PA alone inhibited HT‐29 cell proliferation (*p* < 0.0001). But this inhibitory effect was partially reversed by LPS (*p* < 0.001). OA exhibited dose‐dependent cytotoxicity on HT‐29 cells (*p* < 0.0001), whereas AA and DHA alone had no significant effect on HT‐29 cell proliferation. In the presence of LPS (0.5 ng/mL), OA and AA enhanced HT‐29 cell proliferation compared to their effects alone (*p* < 0.001, *p* < 0.01, respectively), whereas DHA did not exhibit the same effect (Figure 1A,B). Due to poor cell culture conditions observed at fatty acid concentration o ≥ 0.5 mM, subsequent experiments and discussions focused on the effect of fatty acids at 0.1 mM. These findings suggest that the saturated fatty acid PA promotes colon cancer cell proliferation, and this effect is potentiated by LPS. Unsaturated fatty acids (OA, AA, DHA) alone showed no significant impact on colon cancer cell growth. However, OA and AA enhanced HT‐29 cell proliferation in the presence of stimulated gut microbiota, whereas DHA did not.

**FIGURE 2:**
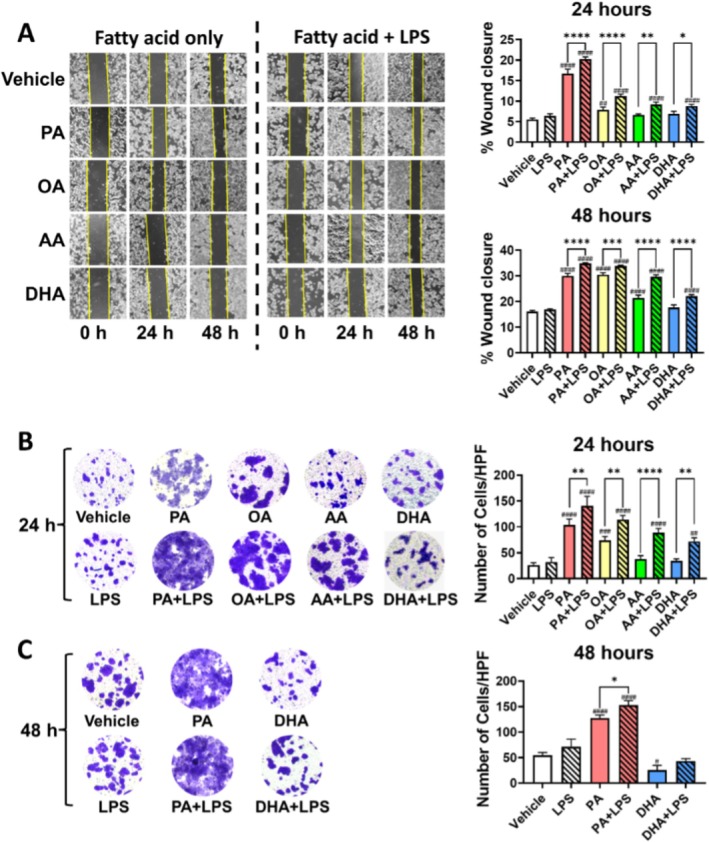
Effects of Fatty Acid and LPS on the Migration and Invasion of Colon Cancer Cells In Vitro. HT‐29 colon cancer cells were cultured with fatty acids (PA, OA, AA, DHA; 0.1 mM) and/or LPS (0.5 ng/mL) for 24 h. (A) Cell migration was assessed using a wound healing assay. Cell invasion was evaluated using the transwell assay following (B) 24 h or (C) 48 h exposure to fatty acids and/or LPS. Representative images and corresponding histograms are presented. Data are expressed as the mean ± SD; *n* = 3 samples per group. ^#^ *p* < 0.05; ^##^ *p* < 0.01; ^###^ *p* < 0.001; ^####^ *p* < 0.0001 compared with vehicle groups and *p < 0.05; **p < 0.01; ***p < 0.001; ****p < 0.0001 by 1‐way ANOVA with Dunnett's multiple‐comparisons test and Bartlett's test of equal variances.

Following 24 h of exposure to fatty acids at 0.1 mM, both PA and OA significantly promoted the migration of HT‐29 cells, with PA exhibiting a stronger effect than OA (*p* < 0.0001). This migratory effect was further enhanced in the presence of LPS. Neither AA nor DHA alone promoted HT‐29 cell migration under the same conditions. However, in the presence of LPS, both AA and DHA significantly enhanced HT‐29 cell migration compared to the vehicle group. Following 48 h of exposure, increased cellular migration was observed across all groups. The pro‐migratory effects of PA and OA were notably stronger than those of AA. Notably, prolonged exposure significantly enhanced the migratory effect of OA. In contrast, DHA alone did not facilitate HT‐29 cell migration, even with extended exposure time. Similarly, the presence of LPS enhanced the migratory capacity induced by all fatty acids (Figure 2A).

Cellular invasion was evaluated using transwell assay. Consistent with the migration assay results, PA and OA alone significantly facilitated HT‐29 cell invasion, with PA showing a stronger effect than OA (*p* < 0.0001). In contrast, AA and DHA did not promote invasion following 24 h of exposure. In the presence of LPS, all the fatty acids exhibited enhanced abilities to promote HT‐29 cell invasion compared to their effect alone (Figure 2B). These results indicate that PA exhibited the strongest pro‐invasion effect among all fatty acids, which was markedly different from DHA. Thus, subsequent experiments focused on the contrasting effects of PA and DHA. Following 48 h of PA exposure, cellular invasion increased significantly (*p* < 0.0001), and this effect was further amplified in the presence of LPS (*p* < 0.05). Conversely, DHA inhibited HT‐29 cell invasion compared to the vehicle group (*p* < 0.05). However, in the presence of LPS, the inhibitory effect of DHA on cellular invasion was attenuated (Figure 2C).

**FIGURE 3:**
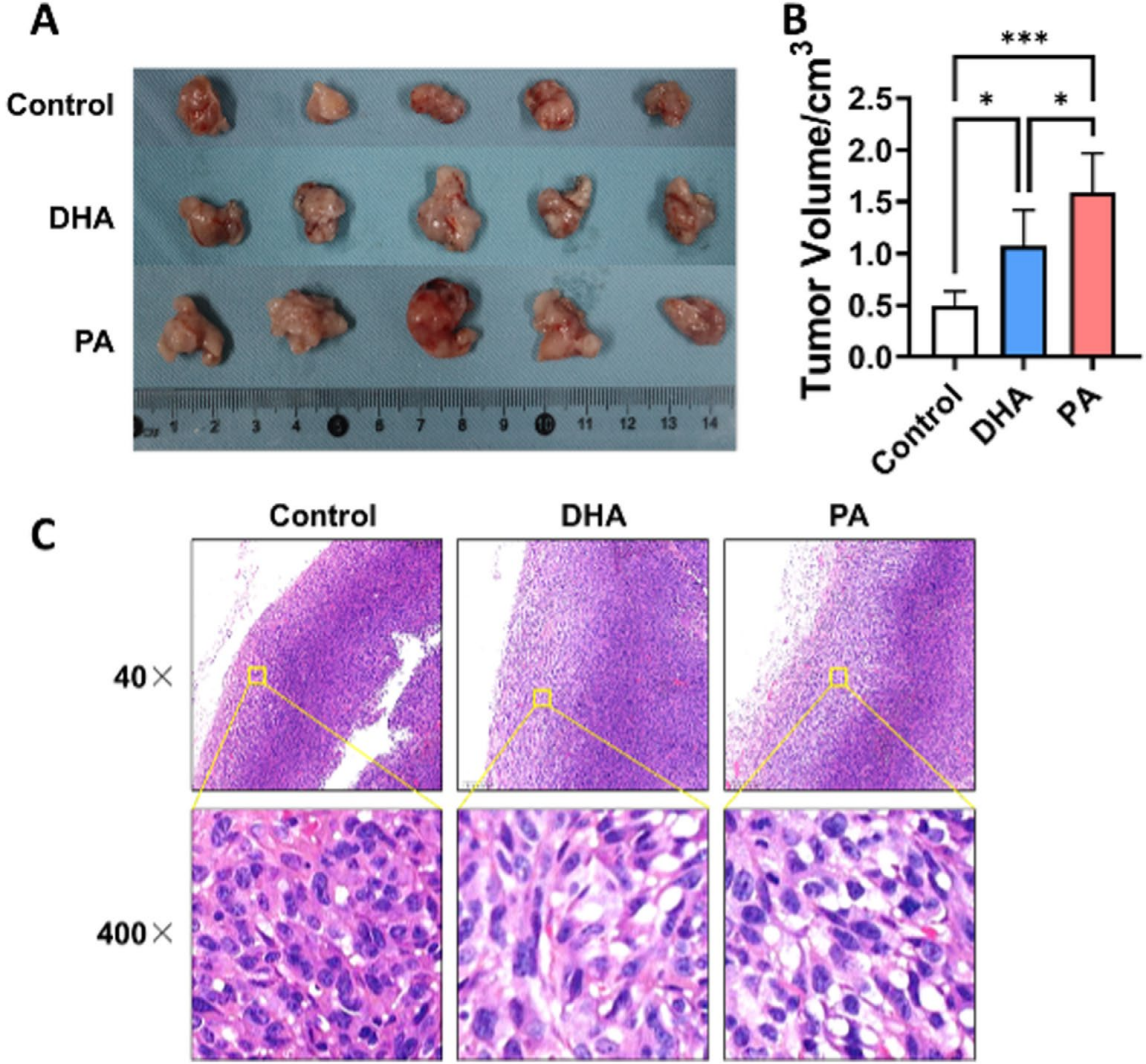
Fatty Acids Promote Tumor Growth and Intracellular Fatty Acid Uptake In Vivo. A tumor‐bearing model was established in C57BL/6 mice through subcutaneous implantation of MC38 colon cancer cells. The mice were randomly assigned to three dietary groups: Regular diet, high‐DHA diet and high‐PA diet. (A) Represent photographs of subcutaneous tumors from each dietary group. (B) Histogram showing tumor volumes across the different groups. (C) Representative images of tumor pathological sections stained with hematoxylin and eosin (H&E). Data are presented as the mean ± SD; *n* = 5 samples per group. *p < 0.05; **p < 0.001 by 1‐way ANOVA with Dunnett's multiple‐comparisons test and Bartlett's test of equal variances.

To further investigate the in vivo effect of PA and DHA, a subcutaneous tumor implantation model was established in C57BL/6 mice using MC38 colon cancer cells. The mice were then randomly divided into three groups and fed different diets. After 12 days of feeding, both the high‐DHA‐diet (0.49 ± 0.14 cm^3^) and high‐PA‐diet (1.07 ± 0.35 cm^3^) promoted tumor growth compared to the control group (1.59 ± 0.37 cm^3^). Notably, the high‐PA‐diet exhibited a stronger tumor‐promoting effect compared to high‐DHA‐diet (Figure 3A,B).

Pathological analysis revealed shallowly stained areas at the tumor margins in both the high‐DHA and high‐PA‐diet groups compared to the control group. High‐resolution images showed cytoplasmic vacuoles, indicating excessive fatty acid uptake by cancer cells (Figure 3C). These findings suggest that colon cancer cells preferentially take up PA over DHA, as evidenced by the greater abundance of cytoplasmic vacuoles in the PA group compared to the DHA group.

### 2.3 Lipopolysaccharide (LPS) Enhances the Uptake of Saturated Fatty Acids In Vitro

**FIGURE 4:**
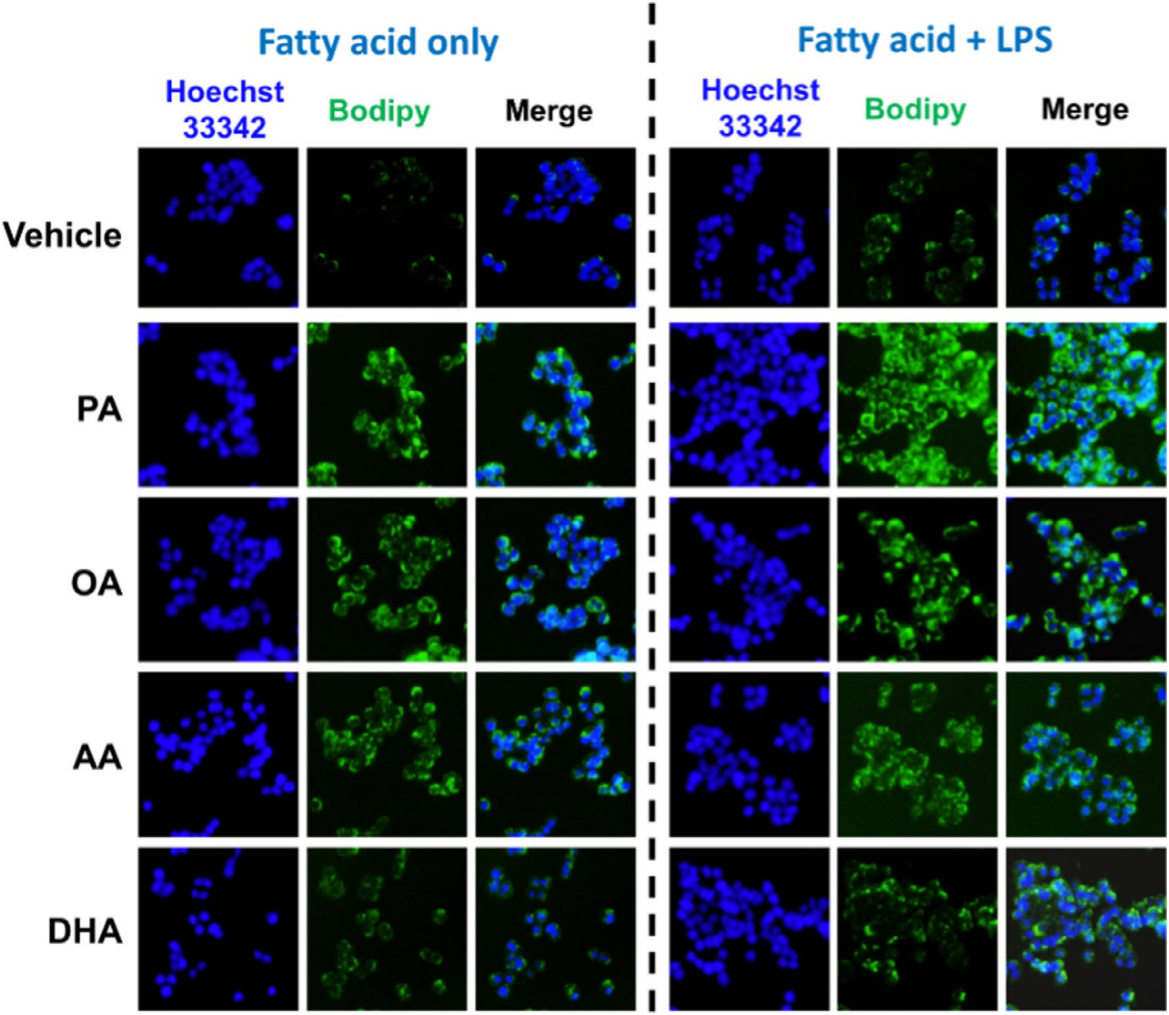
LPS Enhances Fatty Acid Uptake in Colon Cancer Cells In Vitro. HT‐29 colon cancer cells were cultured with fatty acids (PA, OA, AA, DHA; 0.1 mM) and/or LPS (0.5 ng/mL) for 24 h. Fatty acid uptake was assessed using Bodipy staining. Hoechst 33342 staining was used to visualize cellular nuclei. Representative images of Bodipy and Hoechst 33342 staining are presented.

In vivo experiments revealed varying degrees of fatty acid uptake by colon cancer cells. To explore these differences, Bodipy staining was conducted on HT‐29 cells following treatment with different fatty acids. HT‐29 cells demonstrated slightly higher uptake of PA compared to OA, AA, and DHA. DHA uptake was significantly lower compared to the other fatty acids. In the presence of LPS, the uptake of all fatty acids was markedly increased, with the most pronounced effect observed for PA (Figure 4). The differential uptake of these fatty acids may partially explain their varying tumor‐promoting effects. It is likely that LPS enhanced the tumor‐promoting effects of fatty acids by increasing their cellular uptake.

### 2.4 Hyperlipemia Correlates With Elevated Indoleamine 2,3‐Dioxygenase‐1 (IDO1) Expression in Colorectal Cancer

**FIGURE 5:**
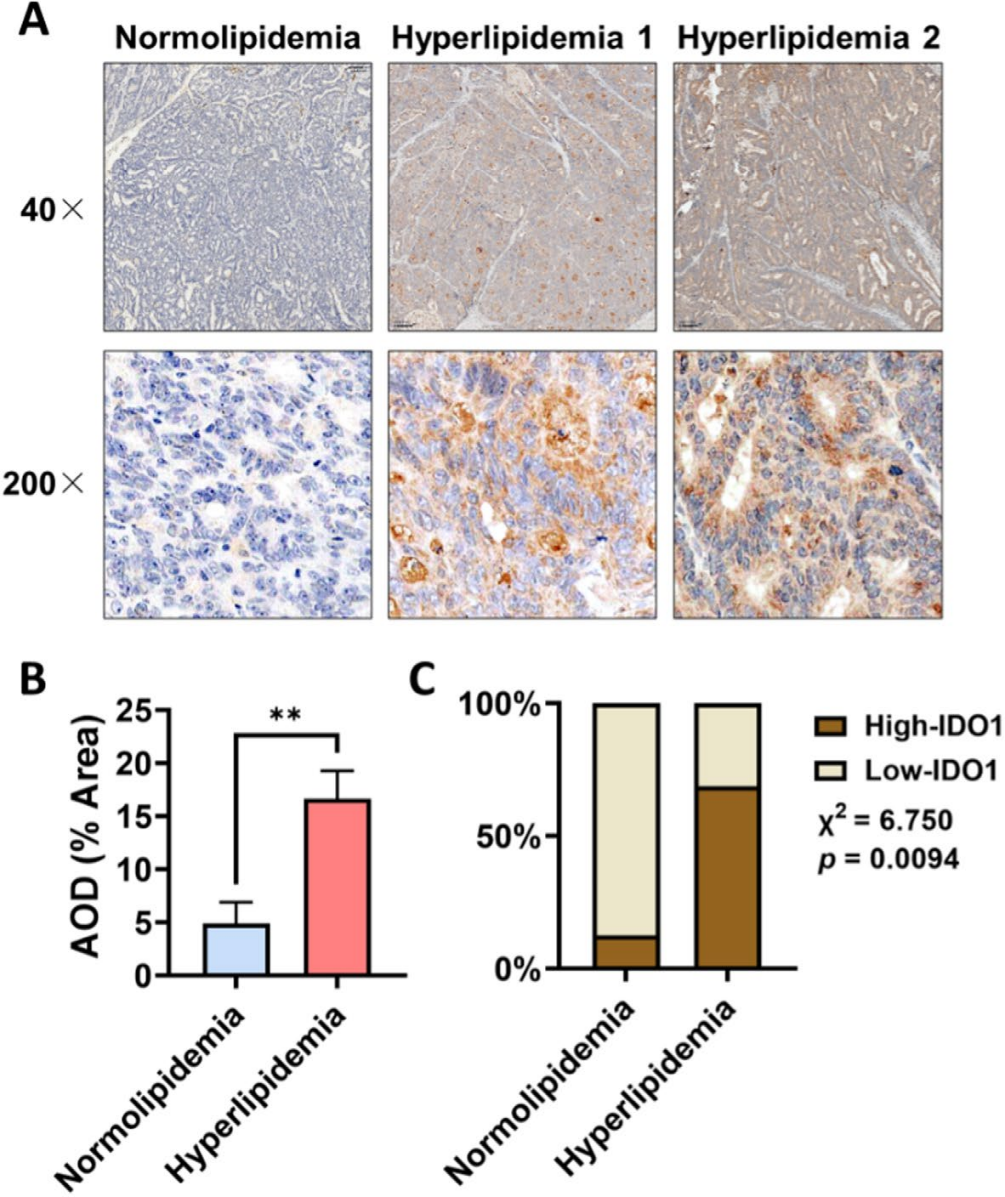
IDO1 Expression Correlates with Blood Lipid Levels in Colorectal Cancer Patients. Colorectal cancer patients were divided into a normolipidemia group (*n* = 8) and a hyperlipidemia group (*n* = 16). Immunohistochemistry (IHC) was conducted to evaluate IDO1 expression levels. (A) Representative IHC images are shown. (B) Histogram showing the average optical density (AOD) of IDO1 expression. Data are presented as the mean ± SD; **p < 0.01 by unpaired students' *t*‐test of equal variances. (C) Contingency analysis showing the percentage of patients high or low IDO1 expression.

To investigate the correlation between IDO1 expression and blood lipid levels in CRC, participants were divided into normolipidemia and hyperlipidemia groups. Immunohistochemistry (IHC) was conducted on pathological tissue sections from CRC patients to evaluate IDO1 expression levels (Figure 5A). In the normolipidemia group, the average optical density (AOD) was 4.871 ± 5.718, significantly lower than the hyperlipidemia group, which had an AOD of 16.63 ± 10.55 (*p* < 0.01) (Figure 5B). Tissue sections were further stratified into high and low IDO1 expression groups on H‐scores (Figure 5C). A chi‐square test revealed a statistically significant association between IDO1 expression and blood lipid levels (*p* < 0.01). These results indicate that IDO1 expression in tumor tissue is significantly elevated in patients with higher blood lipid levels.

### 2.5 A High‐Fatty‐Acid Diet Increases IDO1 Expression in Subcutaneous Tumors in Mice

**FIGURE 6:**
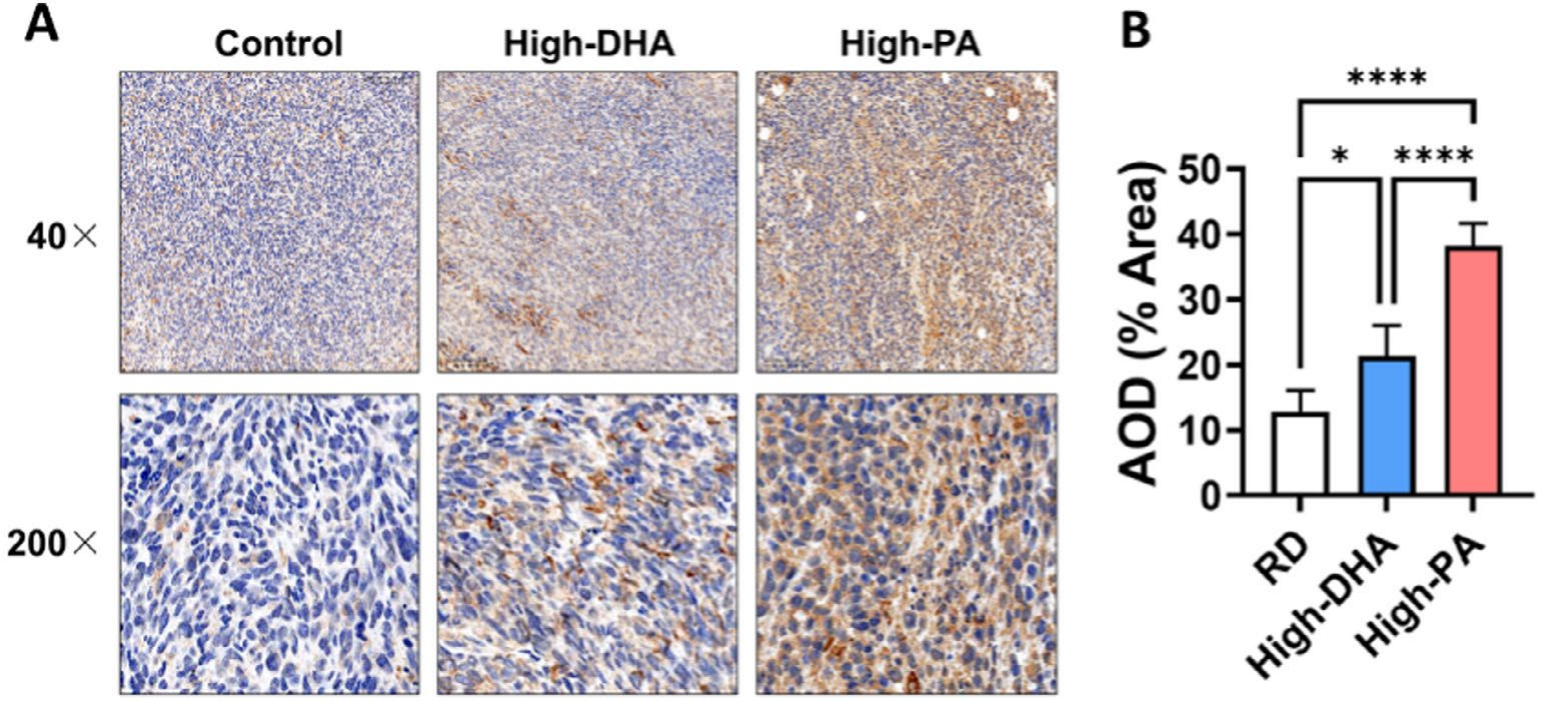
High‐PA and High‐DHA Diets Increases IDO1 Expression in Tumors of Mice. A tumor‐bearing model was established in C57BL/6 mice through subcutaneous implantation of MC38 colon cancer cells. The mice were then randomly assigned to three dietary groups: Regular diet (RD), high‐DHA diet and high‐PA diet. Immunohistochemistry (IHC) was performed to evaluated IDO1 expression levels in tumor tissues. (A) Representative IHC images of IDO1 expression are shown. (B) Histogram depicting average optical density (AOD) of IDO1 expression. Data are presented as the mean ± SD; *n* = 5 samples in each group. *p < 0.05; ****p < 0.0001 by 1‐way ANOVA with Dunnett's multiple‐comparisons test and Bartlett's test of equal variances.

To evaluate the effects of different fatty acids on IDO1 expression, IHC was conducted on pathological sections of subcutaneous tumors from mice fed either a regular diet (RD), a high‐palmitic‐acid diet (high‐PA) or a high‐docosahexaenoic‐acid diet (high‐DHA). IDO1 expression levels were elevated in subcutaneous tumors from mice in both the high‐PA and high‐DHA diet groups, with a significantly greater increase observed in the high‐PA diet group compared to the high‐DHA group (*p* < 0.0001) (Figure 6A,B). These findings suggest that dietary fatty acids, particularly palmitic acid, may upregulate IDO1 expression in tumor tissues, potentially contributing to a pro‐tumorigenic effect.

### 2.6 Saturated Fatty Acids Promote Activation of the IDO1/AHR/NF‐κB Pathway in Colon Cancer Cells

IDO1 is known to activate the aromatic hydrocarbon receptor (AhR) indirectly through Kynurenine (Kyn). IDO1 can also upregulate the PI3K/Akt pathway either directly or indirectly through AhR activation, thereby influencing cellular proliferation and survival. Additionally, activation of the PI3K/Akt pathway promotes NF‐κB expression, which subsequently increases IDO1 expression. This forms a positive feedback loop (IDO1‐AhR‐PI3K/Akt‐NF‐κB), which may underlie the mechanism by which fatty acids promote CRC progression.

**FIGURE 7:**
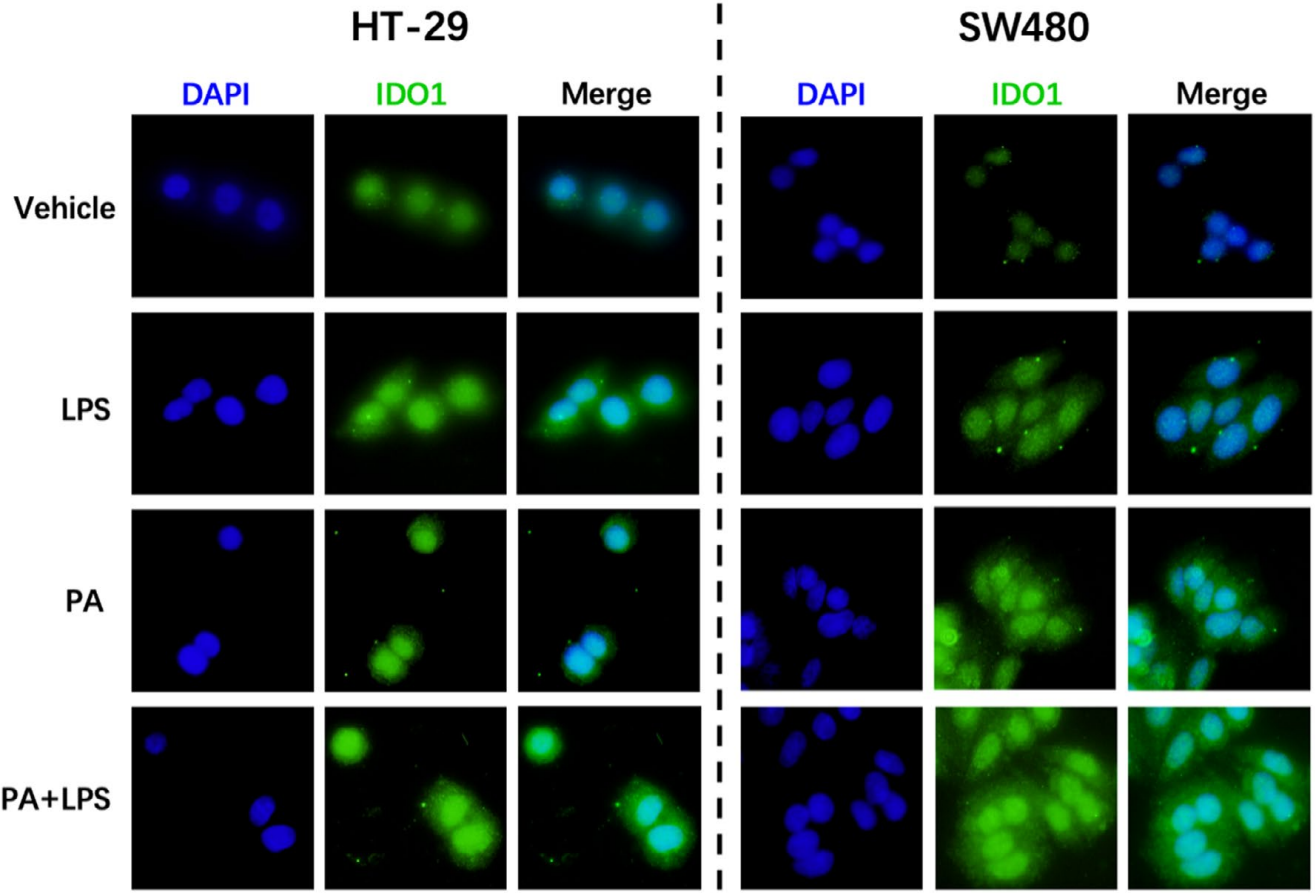
Saturated Fatty Acids and LPS Upregulate IDO1 Expression in Colon Cancer Cells In Vitro. HT‐29 and SW480 colon cancer cells were cultured with PA (0.1 mM) and/or LPS (0.5 ng/mL) for 24 h. IDO1 expression (green) was detected by immunofluorescence staining. Cell nuclei were counterstained with DAPI (blue). Representative images of IDO1 and DAPI staining are presented.

To investigate the role of IDO1 in modulating metabolic pathways in CRC, we treated HT‐29 and SW480 cells with PA, LPS, or their combination. Immunofluorescence analysis revealed that treatment with PA or LPS alone induced a modest increase in IDO1 expression in both cell lines. Notably, the combined treatment of PA and LPS resulted in a pronounced synergistic upregulation of IDO1, as evidenced by a marked intensification of green fluorescent signal in the merged images (Figure 7). This finding demonstrates that the saturated fatty acids cooperate with LPS to potently enhance IDO1 expression in CRC cells in vitro.

**FIGURE 8:**
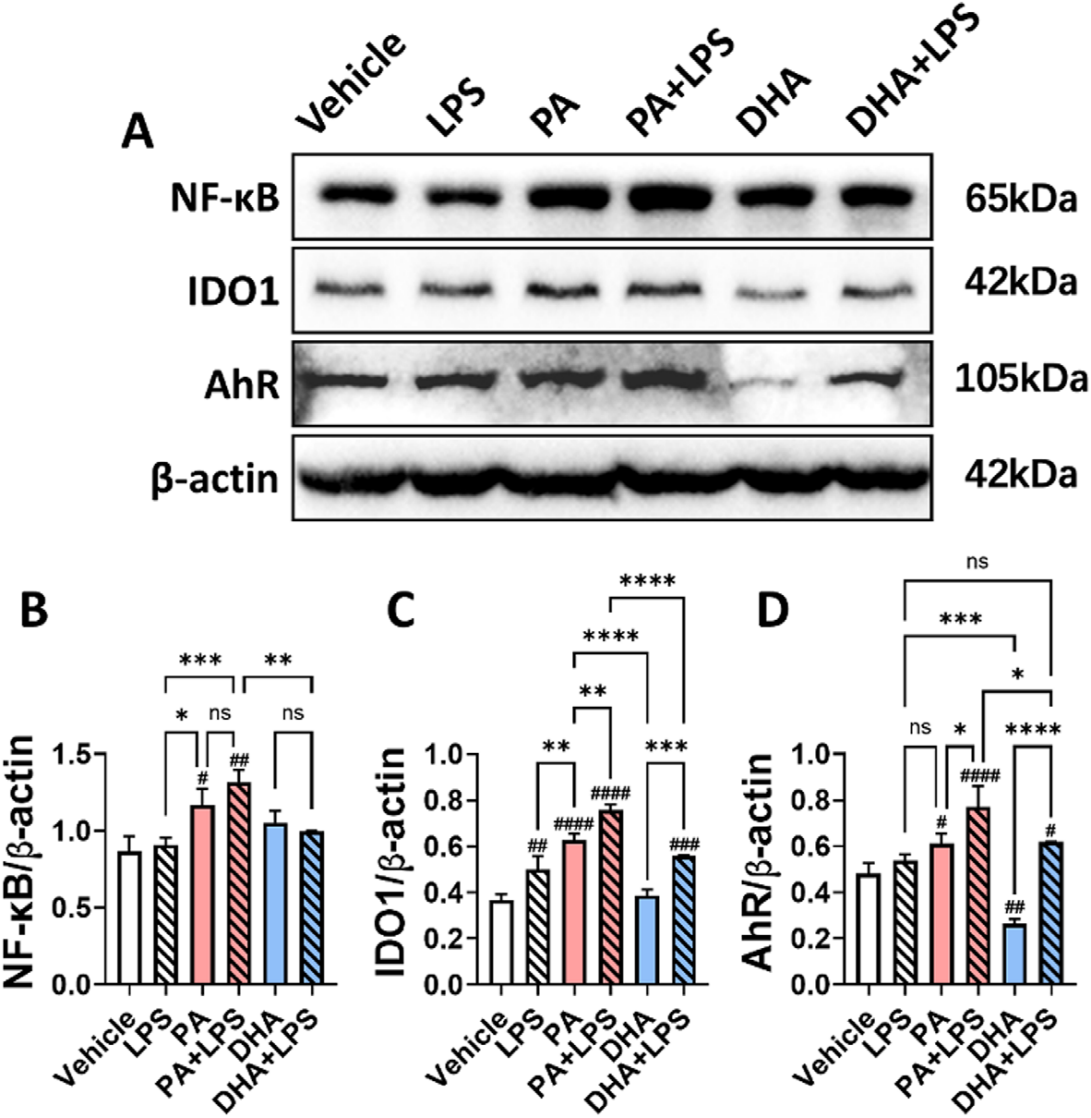
Effect of PA and DHA on the Expression of IDO1, AhR, and NF‐κB Proteins In Vitro. HT‐29 colon cancer cells were cultured with fatty acids (PA, DHA; 0.1 mM) and/or LPS (0.5 ng/mL) for 24 h. Western blot analysis was conducted to assess the expression of NF‐κB, IDO1, and AhR proteins. (A) Representative Western blot images. Semi‐quantitative analysis of the expression levels of (B) NF‐κB, (C) IDO1, and (D) AhR. Protein band intensities were normalized to β‐Actin. Data are presented as the mean ± SD; *n* = 3 samples per. ^#^ *p* < 0.05; ^##^ *p* < 0.01; ^###^ *p* < 0.001; ^####^ *p* < 0.0001 compared with vehicle groups and *p < 0.05; **p < 0.01; ***p < 0.001; ****p < 0.0001 by 1‐way ANOVA with Dunnett's multiple‐comparisons test and Bartlett's test of equal variances.

Western blot analysis was conducted to evaluate the expression of pathway‐related proteins in HT‐29 cells treated with PA (0.1 mM) or DHA (0.1 mM), either alone or in combination with LPS (0.5 ng/mL) for 24 h (Figure 8A). PA and PA + LPS treatments increased NF‐κB expression levels in colon cancer cells (*p* < 0.05, *p* < 001, respectively), whereas DHA alone or DHA + LPS did not have a significantly effect on NF‐κB expression (Figure 8B). PA treatment increase IDO1 expression compared to vehicle group (*p* < 0.0001), and this effect was further enhanced in the presence of LPS (*p* < 0.01). DHA alone did not affect IDO1 expression in colon cancer cells. However, in the presence of LPS, DHA modestly increased IDO1 levels compared to the vehicle group (*p* < 0.001), but the levels remained lower than those observed in the PA + LPS group (*p* < 0.0001) (Figure 8C). Similarly, PA treatment increased AhR expression levels (*p* < 0.05), which were further enhanced in the presence of LPS (*p* < 0.05). In contrast, DHA alone inhibited AhR expression compared to vehicle group (*p* < 0.01). When combined with LPS, DHA increased AhR expression levels compared to the vehicle group (*p* < 0.05), but lower than the PA + LPS group (*p* < 0.05) (Figure 8D).

**FIGURE 9:**
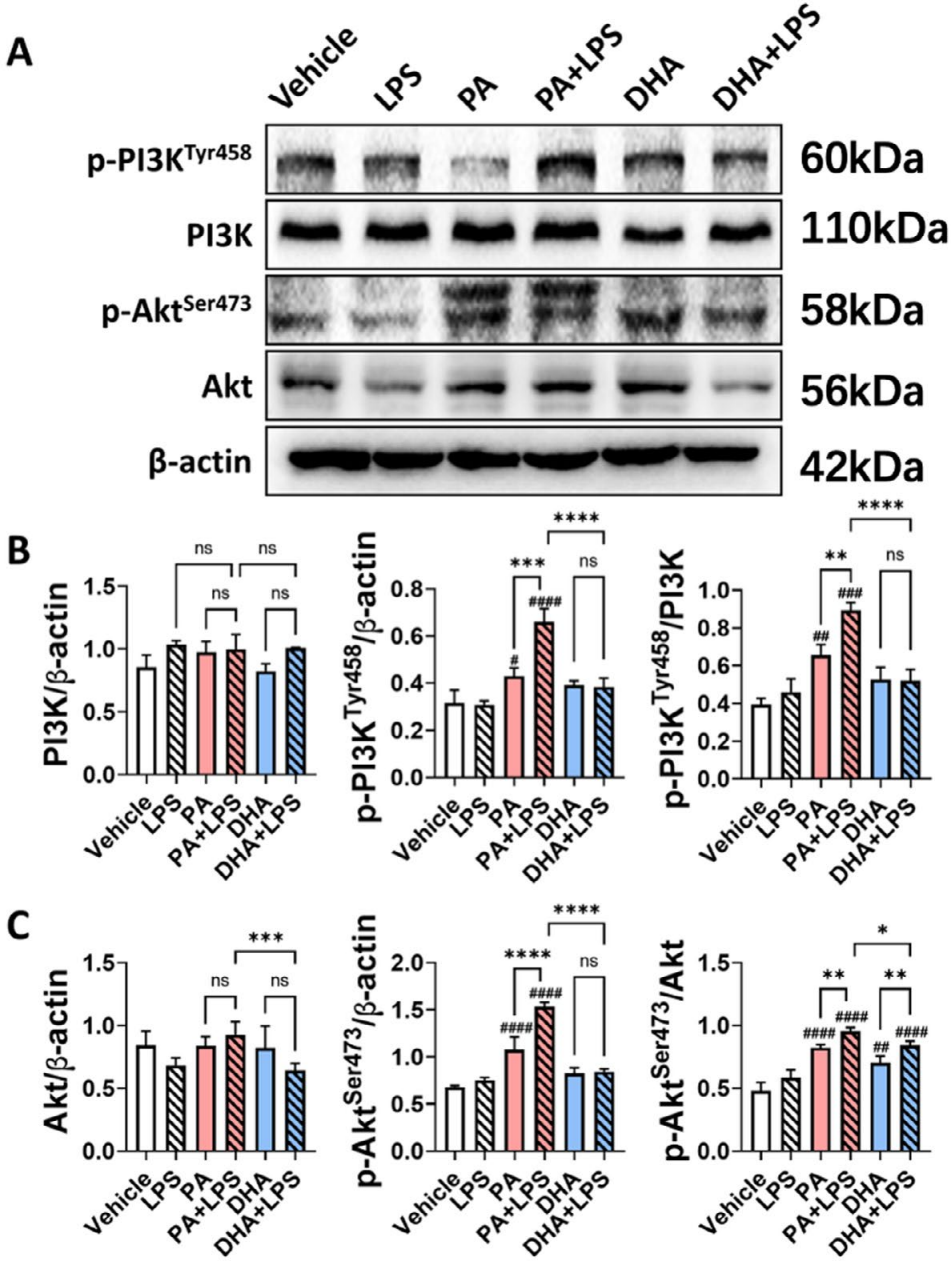
Effect of PA and DHA on PI3K/Akt Pathway Proteins Expression In Vitro. HT‐29 colon cancer cells were cultured with fatty acids (PA, DHA; 0.1 mM) and/or LPS (0.5 ng/mL) for 24 h. (A) Representative Western blot images show the expression levels of p‐PI3K^Tyr458^, total PI3K, p‐Akt^SER473^ and total Akt. Semi‐quantitative analysis illustrates the expression levels of (B) p‐PI3K^Tyr458^ and total PI3K, and (C) p‐Akt^SER473^ and total Akt. Band intensities for each protein were normalized to β‐Actin or their respective total protein (PI3K and Akt). Data are presented as the mean ± SD; *n* = 3 samples per group. ^#^ *p* < 0.05; ^##^ *p* < 0.01; ^###^ *p* < 0.001; ^####^ *p* < 0.0001 compared with vehicle groups and *p < 0.05; **p < 0.01; ***p < 0.001; ****p < 0.0001 by 1‐way ANOVA with Dunnett's multiple‐comparisons test and Bartlett's test of equal variances.

The expression of PI3K/Akt pathway proteins were analyzed to determine their involvement in CRC progression (Figure 9A). Neither PA nor DHA altered the total expression levels of PI3K. PA alone or PA + LPS significantly increased the levels of p‐PI3K^Tyr458^ (*p* < 0.05, *p* < 0.0001, respectively), whereas DHA alone or DHA + LPS had no effect on PI3K^Tyr458^ expression. The ratio of p‐PI3K^Tyr458^ to total PI3K confirmed that PA alone enhanced PI3K phosphorylation (*p* < 0.01), and this effect was further amplified in the presence of LPS (*p* < 0.001). In contrast, neither DHA nor DHA + LPS significantly affect PI3K phosphorylation (Figure 9B). Similarly, neither PA nor DHA altered the total Akt expression levels compared to the vehicle group. However, the DHA + LPS group exhibited lower total Akt levels compared to the PA + LPS group (*p* < 0.001). PA and PA + LPS significantly increased p‐Akt^Ser473^ levels compared to the vehicle (*p* < 0.0001), whereas DHA did not affect p‐Akt^Ser473^ expression. The ratio of p‐Akt^Ser473^ to total Akt indicated that PA significantly increased Akt phosphorylation (*p* < 0.0001), and this effect was further enhanced in the presence of LPS (*p* < 0.01). Notably, PA + LPS exhibited a stronger ability to phosphorylate Akt compared to DHA + LPS (*p* < 0.05) (Figure 9C). The Western blot results suggest that saturated fatty acids activate the IDO1‐AhR‐PI3K/Akt‐ NF‐κB pathway, potentially contributing to their tumor‐promoting effects in CRC progression.

**FIGURE 10:**
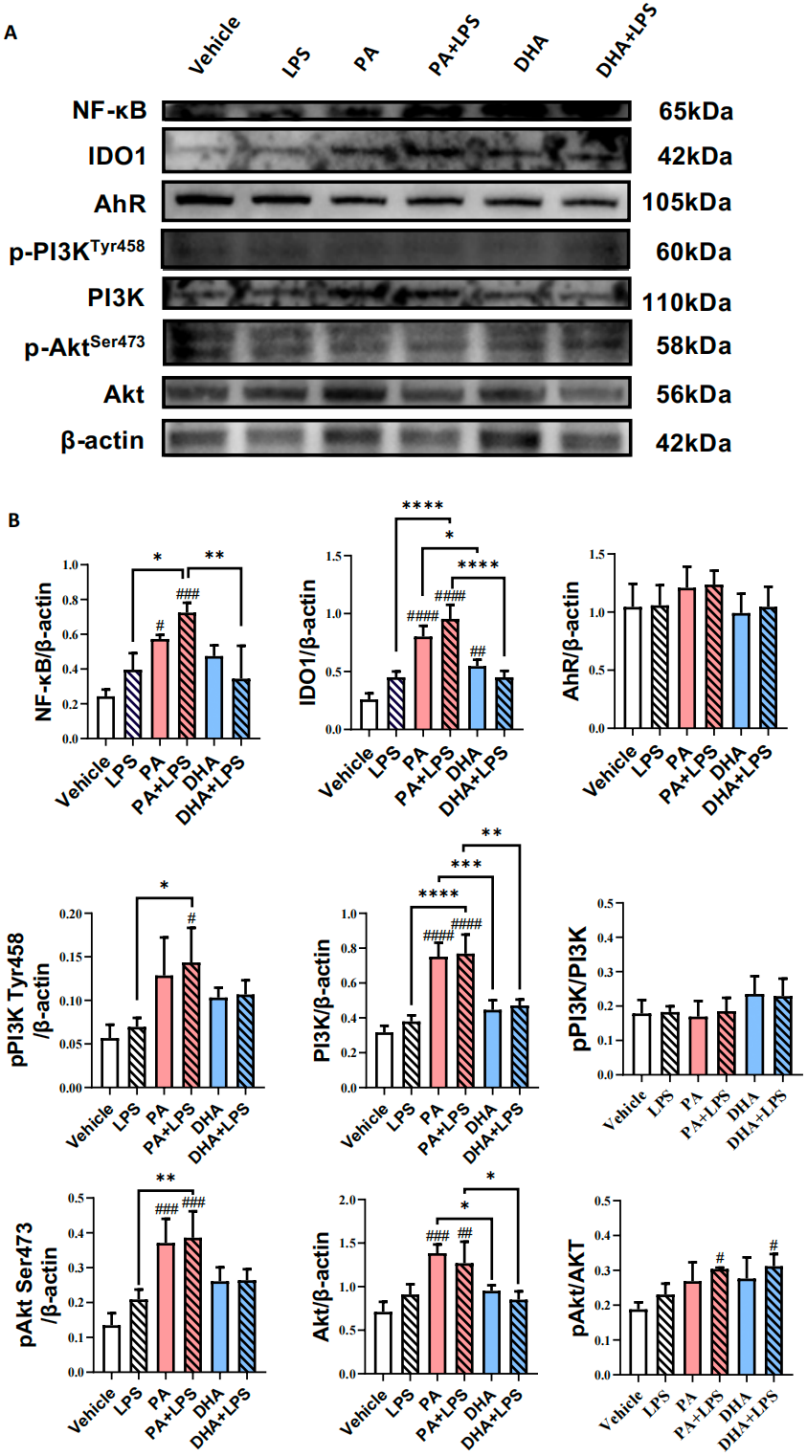
Effect of PA and DHA on IDO1 Pathway Proteins Expression In SW480 CRC cells. SW480 colon cancer cells were cultured with fatty acids (PA, DHA; 0.1 mM) and/or LPS (0.5 ng/mL) for 24 h. (A) Representative Western blot images show the expression levels of NF‐κB, IDO1, AhR, p‐PI3K^Tyr458^, total PI3K, p‐Akt^SER473^ and total Akt. (B) Semi‐quantitative analysis illustrates the expression levels of all proteins. Data are presented as the mean ± SD; *n* = 3 samples per group. ^#^ *p* < 0.05; ^##^ *p* < 0.01; ^###^ *p* < 0.001; ^####^ *p* < 0.0001 compared with vehicle groups and *p < 0.05; **p < 0.01; ***p < 0.001; ****p < 0.0001 by 1‐way ANOVA with Dunnett's multiple‐comparisons test and Bartlett's test of equal variances.

To validate the reliability of our findings, we repeated the in vitro experiments using another colon cancer cell line, SW480, and obtained results consistent with those observed in HT‐29 cells. Treatment with PA alone or in combination with LPS significantly upregulated the expression of key proteins in the IDO1 signaling pathway compared to the vehicle control (*p* < 0.0001). In contrast, DHA alone showed minimal or inhibitory effects on these proteins, and its combination with LPS induced only modest upregulation, which remained significantly lower than that induced by PA + LPS (Figure 10).

### 2.7 IDO1 Expression Increases Over Time

**FIGURE 11:**
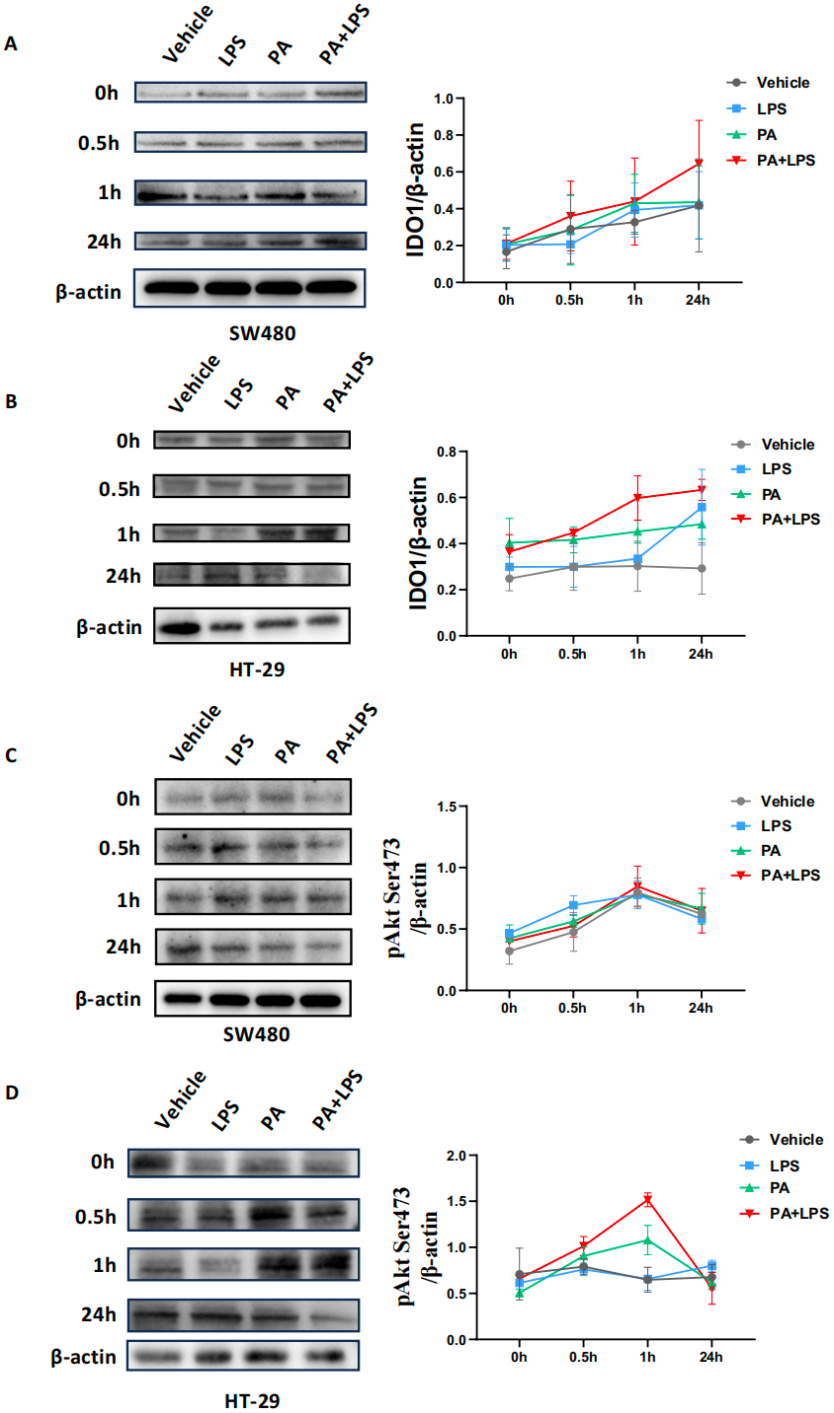
Dynamics of IDO1 and p‐AKT Activation in Response to PA and LPS. SW480 and HT‐29 CRC cells were cultured with PA (0.1 mM) and/or LPS (0.5 ng/mL) for 0, 0.5, 1 and 24 h. (A) Representative Western blot images show the expression levels of IDO1 at 0, 0.5, 1 and 24 h timepoints, and quantitative analysis of IDO1 expression trends in SW480 CRC cells. (B) in HT‐29 CRC cells. (C) Representative Western blot images show the expression levels of p‐Akt^Ser473^ at 0, 0.5, 1 and 24 h timepoints, and quantitative analysis of p‐Akt^Ser473^ expression trends in SW480 CRC cells. (D) in HT‐29 CRC cells.

To determine the dynamic changes in protein expression along the signaling pathway over time, we observed that IDO1 protein levels increased progressively at the measured time points (0, 0.5, 1, and 24 h) in SW480 and HT‐29 CRC cells. Furthermore, the combination of PA + LPS promoted a more pronounced increase in IDO1 expression (Figure 11A,B). Additionally, the expression of p‐Akt^Ser473^ peaked at 1 h after stimulation (Figure 11C,D).

## 3 Discussion

CRC is one of the most prevalent malignant tumors worldwide, with especially rising incidence in China linked to Westernized diets, obesity and reduced physical activity [18]. Evidence strongly correlates high‐fat diets and hyperlipidemia with CRC and its precursor, colorectal adenomas (CRA). Elevated levels of TG, TC, and LDL‐C have been associated with adenoma development and progression, suggesting their potential as biomarkers for high‐risk CRC populations [3, 19]. High‐fat diets promote bile secretion and alter gut microbiota, leading to increased pro‐inflammatory and carcinogenic compounds in the colon. Reduced fiber intake and intestinal motility further prolong exposure to carcinogens. Experimental evidence further demonstrates that high‐fat diets contribute to intestinal neuronal apoptosis, gut microbiota dysbiosis, and intestinal inflammation, all of which are potential mechanisms driving CRC development [20, 21]. Nevertheless, the specific mechanisms underlying high‐fat diet‐induced CRC require further systematic investigation.

In this study, blood lipid levels served as an indirect indicator of dietary fat and fatty acid intake. Analysis of clinical lipid profiles revealed differences between CRC patients and healthy individuals. While TCH, TG, HDL‐C, and LDL‐C are widely utilized in hyperlipidemia diagnosis, other lipid markers such as APOA1, APOB, LPa, sd LDL‐C, and NEFA remain understudied, leading to underutilization of clinical data and obscured potential in CRC screening. Comparing lipid profiles between CRC patients and healthy individuals indirectly confirmed a positive correlation between HFD patterns and CRC incidence. Notably, non‐esterified fatty acid levels were higher in CRC participants, highlighting the potential role of fatty acids in CRC progression.

Altough high‐fat diets are abundant in diverse fatty acids, their specific roles in promoting CRC remain unclear. Literature indicates that SFAs and Ω‐6 PUFAs may promote tumorigenesis, while Ω‐3 PUFAs exhibit inhibitory effects [22, 23]. Although serum MUFAs are negatively correlated with colon cancer incidence, the role of prominent MUFAs like oleic acid remains unclear, with some studies suggesting they promote cancer cell behaviors via signaling pathways, though evidence is inconclusive [24] [25]. Recent studies, consistent with our findings, demonstrate distinct pro‐tumor effects of specific fatty acids: PA promotes proliferation and inflammation [26]; OA alters membrane lipid composition and cellular signaling [27, 28]; and AA facilitates tumor progression through pro‐inflammatory mediators [29, 30]. In contrast, DHA shows anti‐tumor effects such as apoptosis induction and anti‐angiogenesis [31, 32]. The impact of these fatty acids may vary based on individual differences and environmental factors.

In our study, prolonged exposure to PA enhanced colon cancer cell migration and invasion, while DHA inhibited tumor progression. Although a short‐term high‐DHA diet promoted subcutaneous tumor growth in vivo within 12 days, the long‐term effects of high‐fat diets on CRC remain uncertain, particularly given the absence of LPS in the subcutaneous tumor microenvironment compared to the intestine. Among the four representative fatty acids tested, PA demonstrated the strongest pro‐tumor effects both in vitro and in vivo. Other fatty acids—OA, AA, and DHA—did not significantly promote tumor progression alone, though OA and AA exhibited pro‐tumor activity in the presence of LPS. Focusing on low‐dose treatment (0.1 mM) to avoid cytotoxicity, we found that fatty acid uptake varies by type and correlates positively with pro‐tumor potential. LPS enhanced the uptake of all fatty acids, especially PA. This suggests a mechanism possibly involving endocytosis‐mediated LPS internalization, as previously observed in enteric neurons, indicating that fatty acid‐driven carcinogenesis may depend on cellular uptake efficiency, with greater consumption leading to stronger tumor‐promoting effects [33].

Lipopolysaccharide (LPS), a byproduct of the gut microbiota, is commonly employed in basic experiments to simulate the gut microbiota environment [34]. CRC patients often exhibit gut dysbiosis, marked by significant shifts in microbial composition compared to healthy individuals [35]. Diet profoundly shapes the gut microbiota's structure and function, with high‐fat diets notably increasing Firmicutes and reducing Bacteroidetes and Lactobacillus abundances—changes also seen in hyperlipidemia [4] [36]. Beyond structural alterations, high‐fat diets disrupt metabolic functions and are established risk factors for CRC and its liver metastasis, partly through promoting dysbiosis [37]. These dietary‐induced microbial changes lead to the secretion of carcinogenic metabolites and pro‐inflammatory agents. LPS contributes to CRC progression through distinct mechanisms: extracellular LPS binds Toll‐like receptor 4 (TLR4) on colon cancer cells, activating NF‐κB and JNK pathways to enhance migration and invasion [38], while intracellular LPS triggers inflammatory signaling via NOD‐like receptors (NLRs), further aiding tumor progression [39]. LPS and fatty acids act synergistically, especially by elevating intracellular LPS levels. LPS binding to CD14 facilitates its interaction with the TLR4/MD2 complex, initiating NF‐κB activation [40]. Additionally, PA enhances LPS binding affinity [41], underpinning the strong pro‐tumor effects observed in PA + LPS co‐treatment.

IDO1, the rate‐limiting enzyme in tryptophan catabolism, produces Kyn, an AhR ligand that activates the PI3K/Akt pathway and upregulates NF‐κB [42]. High‐fat diets or obesity indirectly increase IDO1 expression, contributing to pathologies such as neuroinflammation, vascular smooth muscle cell apoptosis, and insulin resistance [43, 44, 45]. In CRC, IDO1 promotes proliferation and inhibits apoptosis via Kyn‐mediated PI3K‐Akt activation [46]. It is also linked to immune suppression [47, 48] and therapeutic resistance [49, 50], and is generally associated with CRC initiation, progression and poor clinical outcomes [51]. Nevertheless, the role of IDO1 upregulation in high‐fat diets‐driven CRC remains unclear.

Our results showed that higher IDO1 expression in CRC patients with hyperlipidemia and in tumor‐bearing mice fed high‐fat diets correlates with fatty acid pro‐tumor effects. Mechanistically, PA activates the IDO1‐AhR‐PI3K/Akt‐NF‐κB pathway, enhanced by LPS, while DHA suppresses AhR and only mildly activates Akt. Since both PI3K/Akt and NF‐κB promote CRC progression, this pathway likely mediates the tumor‐promoting effects of high‐fat diets.

In summary, studies integrating clinical data with basic experimental research have confirmed that high‐fat diets promote the progression of CRC involving the IDO1‐AhR‐PI3K/Akt‐NF‐κB signaling pathway. The possible process is described as follows: Fatty acids in a high‐fat diet, mediated by gut microbiota, upregulate the expression of IDO1, leading to increased production of endogenous Kyn. Kyn then activates the AhR, which translocates to the cell nucleus and binds to the promoter region of the PI3K/Akt gene. Activation of the PI3K/Akt pathway subsequently enhances both the expression and DNA‐binding capacity of NF‐κB, thereby facilitating the progression of CRC. Although the present findings suggest that the IDO1‐AhR‐PI3K/Akt‐NF‐κB pathway is associated with high‐fat‐diet‐induced CRC progression, further intervention studies targeting this pathway are needed to elucidate the underlying mechanism.

This study has clarified that specific fatty acids exert distinct effects on CRC progression and emphasizes the strong pro‐tumor potential of saturated fatty acids. Reducing fatty acid intake, particularly of saturated fatty acid, along with weight management is recommended to help prevent CRC development. These findings highlight the critical role of IDO1 as a potential biomarker for CRC associated with a high‐fat diet, offering novel therapeutic insights and strategies for CRC treatment through dietary management.

## 4 Methods

### 4.1 Participants and Clinical Samples

All the participants were patients who underwent colonoscopy and concurrent serum lipid level testing at the Second Hospital of Shandong University between January 2019 and March 2023. Tumor tissues were collected from a total of 24 patients diagnosed with colorectal cancer at the Department of Colorectal Surgery and the Digestive System Department of the Second Hospital of Shandong University during the same period.

### 4.2 Subcutaneous Tumor Implantation Model in Mice

Colon cancer MC38 cells was cultured and subcutaneously injected into the flank skin of C57BL/6 mice with a concentration of 2 × 10^6^ cells/100ul per mouse. The mice were randomly divided into three groups and fed with different diets for 12 days: regular diet, high‐DHA diet (formulated by adding 70% DHA enriched fish oil to dry food at 1.6 mL/kg, provided DHA content of 0.11%), and high‐PA diet (lard as the primary fat source, contain 8.2% palmitic acid). Subsequently, the mice were euthanized, and tumors were excised for volume measurement and preparation of pathological sections. Tumor volume (V) was calculated using the formula: V = 0.5*L*W^2^. Where L is tumor's longest diameter, and W is the shortest diameter perpendicular to L.

### 4.3 Materials

The following reagents were used: *Eschericha coli* LPS (Solarbio); stock (6 mM) PA, OA, AA and DHA prepared as previously described [52]; IDO1 antibody (Abcam, ab311847), AhR antibody (Proteintech, 67,785–1‐lg), phospho‐PI3K (Tyr458) antibody (Cell Signaling, 4228S), PI3K antibody (Proteintech, 67,121–1‐lg), phosphor‐Akt (Ser473) antibody (Cell Signaling, 4060S), Akt antibody (Cell Signaling, 9272S), anti‐NF‐κB (Proteintech, 10,745–1‐AP), Biodipy 493/503 lipid stain (Glpbio, GC42959).

### 4.4 Cell Culture

Colon cell line HT‐29, SW480 and MC38 was obtained from Shanghai Fuheng Biology Science and Technology Company LTD. The cells were cultured in high‐glucose Dulbecco's Modified Egale Medium (H‐DMEM, Gibco) supplemented with 10% fetal bovine serum (FBS, Gibco) and 1% Penicillin–Streptomycin (Gibco, 15,070,063)in a humidified tissue culture incubator with 5% CO2 at 37°C.

### 4.5 Cell Viability Assay

HT‐29 cells were cultured in 96‐well plates at 37°C and treated for 24 h with LPS (0.5 ng/mL) and/or BSA‐conjugated fatty acids, including PA (0.1–0.5 mM), OA (0.1–0.5 mM), AA (0.1–0.5 mM), and DHA (0.1–0.5 mM). The Cell Counting Kit‐8 (Beytime, C0038) was used to assess cell viability percentages following the manufacturer's instructions.

### 4.6 Wound Healing Assay

HT‐29 cells were seeded in 6‐well plates at a density of 5 × 10^5^ cells per well. When the cells reached 90% confluence, a linear scratch was made in the monolayer using a sterile 200 μL pipette tip. The cells were subsequently treated with various BSA‐conjugated fatty acids, including PA, OA, AA, and DHA at a concentration of 0.1 mM for 24‐48 h. In parallel, cells were treated with LPS (0.5 ng/mL) alone or in combination with fatty acids. The closure of the scratch area was photographed using an inverted microscope. The scratch width was measured using ImageJ software, and the percentage of wound closure was calculated.

### 4.7 Transwell Invasion Assay

The upper chamber was precoated with 50 μL of Matrigel (BD Biosciences) diluted to a final concentration of 1 mg/mL. The lower chamber was filled with 600 μL of DMEM containing 10% FBS as a chemoattractant. Cells were treated with BS‐conjugated fatty acids (PA, OA, AA, DHA; 0.1 mM) for 24 or 48 h. After incubation, non‐invaded cells on the upper membrane surface were removed using a cotton swab. Cells that invaded through the membrane were fixed with 4% paraformaldehyde, stained with 0.1% crystal violet, and photographed under an inverted microscope. The number of invaded cells was quantified in five random fields per well using ImageJ software.

### 4.8 Immunofluorescence

Following treatment, cells were fixed with 4% paraformaldehyde for 15 min at room temperature. IDO1 primary antibody (diluted 1:200 in antibody dilution buffer) was used for 1.5 h at room temperature, followed by incubation with a fluorophore‐conjugated secondary antibody (diluted 1:200) for 1 h in the dark. Lipid droplets were stained using BODIPY 493/503 at a final concentration of 1 μg/mL for 30 min in the dark. Nuclei were counterstained with DAPI. Fluorescent images were captured using a fluorescence microscope.

### 4.9 Western Blotting

Total protein was extracted from HT‐29 and SW480 cells using RIPA lysis buffer (Beyotime, P0013B) supplemented with protease and phosphatase inhibitors. Protein concentrations were determined using the BCA Protein Assay Kit (Beyotime, P0012). Equal amounts of protein were separated by SDS‐PAGE and transferred onto PVDF membranes (Millipore, IPVH00010). The membrane was blocked and incubated with primary antibodies and HRP‐conjugated secondary antibodies. Subsequently, protein bands were visualized using enhanced chemiluminescence (ECL).

Reagents (Beyotime, P00185) and captured using a gel imaging system. Band intensities were quantified using ImageJ software.

### 4.10 Immunohistochemistry

Tumor tissues were fixed in 4% paraformaldehyde for 24 h, embedded in paraffin, and sectioned into 4 μm‐thick slices. After antigen retrieval, sections were incubated with primary antibodies against IDO1 (1:200, Abcam) overnight at 4°C. Subsequently, sections were incubated with HRP‐conjugated secondary antibodies for 30 min at room temperature. Staining was developed using 3, 3′‐diaminobenzidine (DAB; Dako) and counterstained with hematoxylin. The average optical density (AOD) of immunohistochemical staining was quantified using ImageJ software.

IDO1 is primarily expressed in the nucleus and cytoplasm of tumor cells. Under microscopic observation, staining in either the nucleus or cytoplasm is considered positive. The staining results are scored based on staining intensity (0–3 points) and the percentage of positively stained tumor cells (0–4 points). Intensity scoring is as follows: no staining, 0 points; weak staining, 1 point; moderate staining, 2 points; strong staining, 3 points. Percentage scoring is as follows: < 5%, 0 points; 5%–25%, 1 point; 26%–50%, 2 points; 51%–75%, 3 points; > 75%, 4 points. The final score is obtained by multiplying the intensity and percentage scores, with scores < 6 classified as low expression and scores ≥ 6 classified as high expression. All results were independently evaluated by two pathologists in a double‐blind manner, and discrepancies were resolved through review by a senior pathologist.

### 4.11 Statistical Analysis

All statistical analyses were performed using SPSS software (version 19.0, IBM) or GraphPad Prism (version 9.5). Data were expressed as mean ± standard deviation (SD) from at least three independent experiments. Group comparisons were conducted using one‐way analysis of variance (ANOVA) followed by Tukey's post hoc test for multiple comparisons. For two‐group comparisons, an unpaired two‐tailed Student's *t*‐test was applied. *P*‐values less than 0.05 were considered statistically significant. Graphs were generated using GraphPad Prism.

### 4.12 Study Approval

All animal studies and the human studies were approved by the Medical Ethics Committee of the Second Hospital of Shandong University in accordance with relevant ethical guidelines. Written informed consent was obtained from all participants prior to study enrollment.

## Author Contributions

**Shulin Zhang:** original draft writing, data collection, experimental work, and data analysis. **Jing Zhang:** experimental work and data analysis. **Jiaqi Chen:** data collection. **Abdel Nasser B. Singab:** manuscript writing. **Guang Yang:** experimental work. **Jiaxuan Li:** data collection. **Peiyao Li:** experimental work. **Songlin Wu:** experimental work. **Di Zhao:** experimental work. **Junhua Sun:** data collection. **Lifen Gao:** experimental design supervision. **Guimei Lin:** data analysis supervision. **Guanying Yu:** manuscript writing supervision. **Yanfeng Iv:** experimental design supervision. **Hongbo Wang:** manuscript writing supervision. **Lin Yuan:** data analysis supervision. **Lan Ye:** experimental design, data analysis, and manuscript writing supervision. All authors reviewed and approved the final manuscript.

## Funding

This work was supported by the Shandong Medical Association Qilu Medical Special Project, YKH2022K02112 the Cultivation Fund of the Second Hospital of Shandong University, 2023JX16. Natural Science Foundation of Shandong Province, ZR2021MH104. Science Fund for Distinguished Young Scholars of Shandong Province, tsqn201909178.

## Conflicts of Interest

The authors declare no conflicts of interest.

## Data Availability

The data that support the findings of this study are available from the corresponding author upon reasonable request.
